# Test- Retest Reliability of the Thai Version of the Mild Behavioral Impairment-Checklist (MBI-C)

**DOI:** 10.1093/geroni/igaf122.3058

**Published:** 2025-12-31

**Authors:** Nussaba Sompanich, Ross Andel, Arunya Tuicomepee, Phot Dhammapeera

**Affiliations:** Chulalongkorn University, Bangkok, Krung Thep, Thailand; Arizona State University, Arizona State University, Arizona, United States; Chulalongkorn University, Bangkok, Krung Thep, Thailand; Chulalongkorn University, Bangkok, Krung Thep, Thailand

## Abstract

Mild Behavioral Impairment (MBI) is characterized by persistent behavioral symptoms. Unlike mild cognitive impairment, it focuses on changes in behavior, mood, and personality rather than cognitive deficits. Mild Behavioral Impairment-Checklist (MBI-C) is a screening tool for MBI. Respondents answer yes/no and rate severity from 1 (Mild) to 3 (Severe) for 34 items covering changes in motivation, mood/anxiety, impulsivity, abnormal thoughts, and social appropriateness. This study aims to assess MBI-C in Thailand. We double-translated the MBI-C to Thai and examined its test-retest reliability among 73 Bangkok residents over 50 (mean age= 58.94 ± 5.621, 75.3% were women), administering the test twice over a two-week interval. Spearman’s rho correlation yielded an overall test-retest reliability of .423, p < .001. In a post-hoc, per-item assessment, we found that 8 of the 24 items correlated perfectly and 10 items yielded correlations <.10. Overall, we observed lower reliability for items capturing momentary emotions like being anxious or worried about mundane things, erratic behaviors like poor judgement during driving, and emotions directed towards significant others like irritability or stubbornness. Items with high reliability included those reflecting changes in mundane behaviors like being more impatient or having more difficulty staying relaxed. Finally, items with zero variance included more extreme behaviors like sexual disinhibition or new bad habits like excessive alcohol drinking or shoplifting. These results offer novel, general and Thai-culture specific insights into the underlying characteristics of items within the MBI-C scale. The presumed role of Thai collectivism among Thai older adults will also be discussed.

